# An Artificial Intelligence-Based Digital Analysis of Immunolocalization of MMP2 and TIMP2 in the Jejunum of Rats Treated with Calcineurin Inhibitors

**DOI:** 10.3390/biomedicines12091966

**Published:** 2024-08-30

**Authors:** Aleksandra Wilk, Kamila Szumilas, Anna Gimpel, Anna Pilutin, Sylwia Rzeszotek, Karolina Kędzierska-Kapuza

**Affiliations:** 1 Department of Histology and Embryology, Pomeranian Medical University, 70-111 Szczecin, Poland; aleksandra.wilk@pum.edu.pl (A.W.); aniagimpel@gmail.com (A.G.); sylwia.rzeszotek@pum.edu.pl (S.R.); 2 Department of Physiology, Pomeranian Medical University, 70-111 Szczecin, Poland; kamila.szumilas@pum.edu.pl; 3 Department of Gastroenterological Surgery and Transplantology, National Medical Institute, Ministry of Interior Affairs and Administration, Wołoska St. 137, 02-507 Warsaw, Poland; karolina.kedzierska@gmail.com

**Keywords:** artificial intelligence, immunosuppressive drugs, small intestine, jejunum

## Abstract

(1) Background: The main goal of this study was to analyze the morphology of the rat’s jejunum after long-term treatment with calcineurin inhibitor-based immunosuppressive drugs and to investigate their impact on the location of MMP-2 and its inhibitor TIMP-2, as well as the balance between them. (2) Methods: Twenty-four rats were divided into four groups receiving different immunosuppressive regiments. After six months of treatment, the jejunums were collected and analyzed. (3) Results: immunosuppressive drug panels containing calcineurin inhibitors (CNIs) have a negative impact on the morphology and morphometry of the small intestinal wall. These drugs disrupt the MMP-2/TIMP-2 balance. Both CsA and TAC interfere with the synthesis of intercellular matrix components in the connective tissue of the small intestine. Furthermore, tacrolimus appears to disrupt the MMP-2/TIMP-2 balance in the small intestine the most, as the results show the highest difference between MMP-2 and TIMP-2 expression. The results were also confirmed by digital analysis of tissue segmentation. (4) Conclusions: The research conducted in this study is unique because there is limited information available on the direct effects of immunosuppressive drugs on the expression of MMP-2 and their inhibitors in the jejunum. Additionally, this study involves three drugs instead of one, which accurately reflects the panel of drugs used in organ recipients. Our results suggest that immunosuppressive drugs affect morphology and MMP2/TIMP2 immunoexpression; however, further studies are required. AI-based tools provide a reliable analysis of tissue samples, which represents an exciting approach for future histopathological studies. However, the results of the analyses generated by these tools need to be verified by specialists.

## 1. Introduction

Cyclosporin A (CsA) and tacrolimus are two drugs that belong to the calcineurin inhibitors and are commonly used to treat transplant recipients as part of immunosuppressive therapy [1,2,3]. CsA inhibits calcineurin and prevents the translocation of activated nuclear transcription factor T cells, as well as inhibiting the activation of other transcription factors. Its metabolism occurs mainly through the cytochrome P (CYP) 450A3 enzyme system [4]. Tacrolimus, which is around a hundred times more potent than CsA, inhibits cellular activity and the immune response through various mechanisms, with its main effect being the inhibition of calcineurin. It is extensively metabolized in the liver, and its bioavailability varies widely in individual patients. The use of tacrolimus can lead to various side effects, including nephrotoxicity, hypertension, neurotoxicity, diabetes mellitus, hirsutism, cholestasis, hyperuricemia, and gingival hyperplasia [3]. The individual differences in pharmacokinetics and pharmacodynamics of tacrolimus make it challenging to predict the optimal dose and monitor the risk of side effects or treatment failure.

One of the side effects of immunosuppressive drugs is their impact on the structure and function of the small intestine. However, there is still limited information available in the literature, so this paper focuses on this important aspect of immunosuppressive treatment. It discusses markers of abnormal changes in connective tissue, specifically the imbalances of metalloproteinases and their inhibitors [5,6].

Extracellular matrix metalloproteinases (MMPs), also known as Matrix Metalloproteinases, are endopeptidases requiring zinc ions to function. They vary in structure and substrate affinity and are grouped into different categories [7].

Most MMPs are initially released as inactive proenzymes in the extracellular space. The N-terminal domain maintains the enzyme in this inactive form, where the cysteine in the propeptide binds to a zinc atom in the catalytic domain’s active site using thiol groups. The entire setup is stabilized by three histidine molecules in the active site. The initial step in activating metalloproteinases involves the separation of the cysteine from the N-terminal domain, leading to the final separation of the propeptide from the MMP. The C-terminal hemopexin domain, located at the end, is involved in the mobilization of endopeptidase, and through its ability to add inhibitors, it affects the inhibition of this process [8,9,10,11,12,13,14].

It is important to note that matrix metalloproteinases play a role in breaking down the extracellular matrix (ECM) and closely interact with their inhibitors—tissue inhibitors of metalloproteinases (TIMPs). An imbalanced MMP/TIMP ratio can lead to processes related to tumor cell growth, cell movement, inflammation, invasion, metastasis, and angiogenesis [15,16].

Active enzyme inhibition is achieved by non-specific inhibitors (α-2 macroglobulin and α1-antitrypsin) as well as specific inhibitors, which include four endogenous inhibitors of metalloproteinases (TIMPs)—TIMP-1, TIMP-2, TIMP-3, TIMP-4. These inhibitors display varying affinity for MMPs and bind to them in a 1:1 ratio, resulting in a reversible inhibition process. The activation of metalloproteinases can also be regulated during gene transcription, zymogen release, and proenzyme activation, which can be induced by various factors such as cytokines (IL-1, IL-6), growth factors (EGF, FGF, VEGF, PDGF, HGF), tumor necrosis factors (TNF-α, TNF-β), antigens (CD40), free radicals, plasmins, thrombin, urokinase, and nitric oxide [8,17,18,19,20,21].

**Figure 1:**
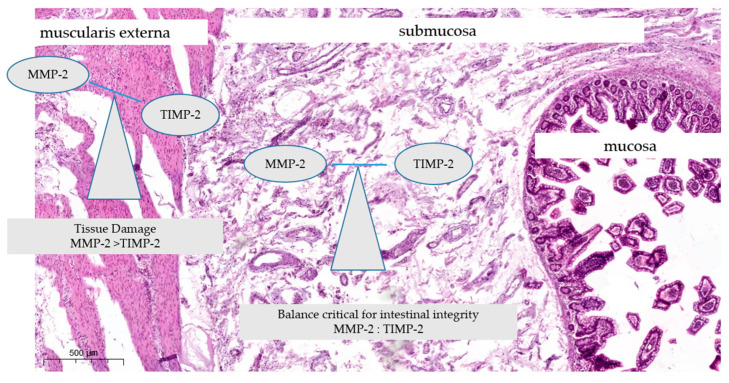
Figure representing the relationship between MMP-2 and TIMP-2 in the intestine: MMP-2 activity: MMP-2 is indicated as degrading the extracellular matrix (ECM) components in the submucosa; TIMP-2 Inhibition: TIMP-2 binds to and inhibits MMP-2, maintaining a balance crucial for tissue integrity; pathological condition: Excessive MMP-2 activity is shown to lead to tissue damage, which can occur when the balance with TIMP-2 is disrupted; original photo: Department of Histology and Embryology, Pomeranian Medical University of Szczecin, Poland.

Calcineurin inhibitors play a crucial role in regulating MMPs and their inhibitors in various diseases, including osteoarthritis and cancer. Calcium-mediated activation of calcineurin promotes MMP-2 activation and decreases TIMP-2 levels, enhancing cellular migration [22]. Calcineurin inhibitors like cyclosporin A can reduce MMP-1 and MMP-3 production while increasing TIMP-1 and collagen II levels in chondrocytes, potentially slowing osteoarthritis progression [23]. MMP-2 activation involves a complex interplay between pro-MMP-2, MT1-MMP, and TIMP-2 [24]. While TIMP-2 inhibits MMPs, it is also essential for MMP-2 activation, making its role in tumor invasion controversial [24]. Calcineurin inhibitors may have side effects on the cardiovascular system, particularly affecting MMP-2 and MMP-9 expression in the heart [25]. The imbalance between MMP-2 and TIMP-2 in the jejunum leads to the destruction of intestinal tissue. Understanding these mechanisms is crucial for developing targeted therapies for various pathological conditions (Figure 1).

Most studies have shown that MMP2 and TIMP2 are involved in the development of colon cancer; however, due to the fact that CNIs affect morphology of many organs, the main goal of this study was to analyze the morphology of the rat’s jejunum after long-term treatment with calcineurin inhibitor-based immunosuppressive drugs and to investigate their impact on the location of MMP-2 and its inhibitor TIMP-2, as well as the balance between them.

## 2. Materials and Methods

### 2.1. Animals

The current study is the continuation of our previous study, and it is conducted on the archival material obtained from the Department of Nephrology, Transplantology, and Internal Diseases of the Pomeranian Medical University Szczecin [18]. Briefly: Material, the fragments of jejunum, were collected from sexually mature male Wistar rats. The experiment was approved by the Local Bioethics Committee for Animal Experiments in Szczecin. In this study, calcineurin inhibitors CsA and Tac, in combination with MMF and a glucocorticosteroid, were administered to the experimental group rats at specific doses. The immunosuppressive drugs were used as follows: (i) CsA (Sandimmun-Neoral)—5 mg/kg b.w./day; (ii) tacrolimus (Prograf)—4 mg/kg b.w./day; (iii) mycophenolate mofetil (Cellcept, Roche)—20 mg/kg b.w./day; (iv) prednisone (Encorton)—4 mg/kg b.w./day. The experimental groups (CMG and TMG) were given immunosuppressive drugs for 6 months [18].

### 2.2. Histochemical Analysis

We performed histological studies to assess the organization of collagen, elastic, and reticulin fibers in the submucosa of the jejunum. The histological slides, which were 3 µm thick, were stained with Hematoxylin and Eosin (HE) and Mallory Trichrome (Bio-Optica Milano, Milan, Italy) to visualize collagen fibers. Additionally, we used silver salt staining (Bio-Optica Milano, Milan, Italy) to visualize reticulin fibers.

### 2.3. Morphometric Analysis

Morphometric analysis was performed for the control group and two study groups. Slide Viewer 2.7 software (3d Histtech, Budapest, Hungary) was used for measurements. We took 30 measurements for each of the 6 histological preparations in each group, measuring the height of the submucosa in micrometers (µm).

### 2.4. Immunohistochemical Analysis

To detect the expression of MMP-2 and TIMP-2, the following mouse antibodies were used: anti-MMP-2 at 1:250(sc-53630; Santa Cruz Biotechnology, Inc., Santa Cruz, CA, USA) and mouse anti-TIMP-2 at 1:250 (sc-21735; Santa Cruz Biotechnology, Inc., Santa Cruz, CA, USA). All antibodies were thinned out with Diluent (Agilent DakoEnVision, Hovedstaden, Denmark). Slides were deparaffinized in three changes in xylene and rehydrated in a graded series of ethanol to distilled water. For antigen retrieval, the slides were immersed in 0.01 M citrate buffer at pH 6.0 and subjected to microwave heating for 10 min. Endogenous peroxidases were blocked by incubation in Dual Endogenous Enzyme Block (Agilent DakoEnVision, Denmark) for 10 min. Afterward, sections were incubated for 30 min at room temperature with the previously mentioned antibodies. Subsequently, sections underwent a 30-minute incubation with labeled polymer (Labeled Polymer HRP; Agilent DakoEnVision, Denmark), followed by a 10-minute incubation with a substrate-chromogen complex containing 3,3′-diaminobenzidine; Agilent DakoEnVision, Denmark), resulting in the formation of a brown precipitate at the antigen site. Negative controls were prepared by excluding the primary antibody. After counterstaining with hematoxylin (Sigma-Aldrich, St. Louis, MO, USA), the specimens were mounted using a permanent mounting medium. Prior to each incubation, tissues were washed twice in PBS for 5 min and subjected to a 5 min TBS bath. Every incubation was conducted in a humid chamber at room temperature. Staining was conducted in compliance with the manufacturer’s protocol. Upon completion of the reaction, the slides were examined under a microscope (Leica DM5000B, Wetzlar, Germany). 

#### Artificial Intelligence (AI)-Related Technology

**Figure 2:**
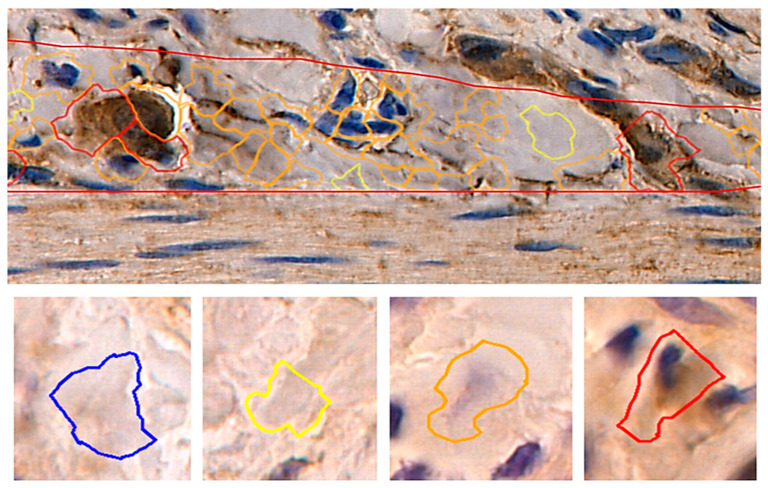
Representative picture of randomly chosen area with four visible strengths of immunoexpression of analyzed enzymes based on AI-related technology. Cell Quant creates a map and marks each observation depending on the strength of immunoexpression of protein: blue circles, negative expression (-); yellow circles, weak expression (+); orange circles, strong expression (++); red circles, very strong expression (+++).

To investigate the intestinal expressions of MMP-2 and TIMP-2, samples were analyzed by the Cell Quant software version 2.3. (3DHISTECH Kft. Budapest, Hungary), which we have used in other research [26]. Due to the fact that Cell Quant is a software that visualizes cytoplasm, nucleus, and/or cell membrane in different levels of intensity, we were able to examine the level of expression of tested proteins. We trained the program to recognize the chosen structures. Cell Quant detected four levels of staining intensity and generated the area and perimeter expression, as well as object intensity of the IHC products (Figure 2). Six slides from each group were analyzed. Since our aim was to analyze tissue areas with the highest quality, small sections were selected and scanned, and the results were then grouped together and extracted. Two independent experts verified the results generated by the Cell Quant software.

### 2.5. Statistical Analysis

Analyses were calculated in the STATA 18 program. Continuous data were presented as medians with interquartile ranges (IQR). Shapiro–Wilk test was applied to evaluate the normal distribution. Since the majority of the data were not normally distributed, the Kruskal–Wallis test and the post hoc Dunn’s test were performed. *p*-value < 0.05 was considered statistically significant.

## 3. Results

### 3.1. Histochemical Analysis

**Figure 3:**
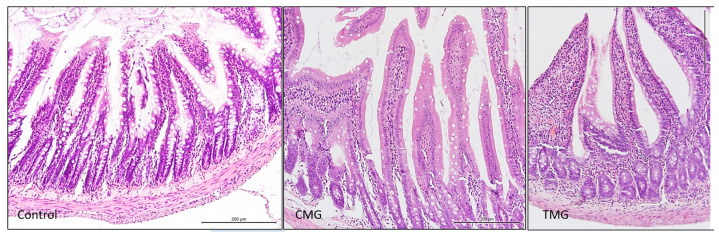
Jejunum stained with HE (objective magnification ×40).

**Figure 4:**
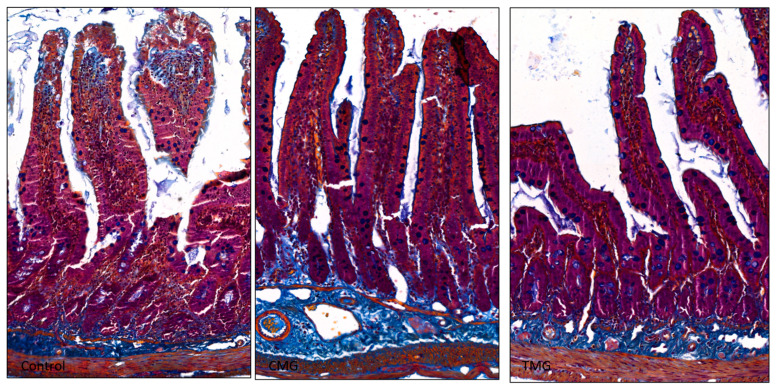
Jejunum stained with Mallory Trichrome (objective magnification ×40).

**Figure 5:**
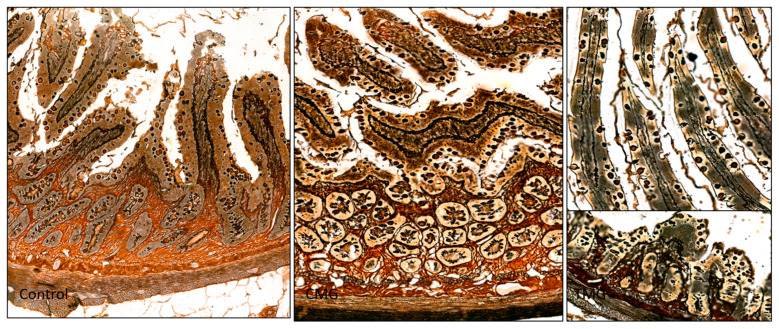
Jejunum stained with silver salts (objective magnification ×40).

The results of the histochemical analysis indicated that the intestinal wall structure was normal in the control group. The collagen fiber bundles in the submucosa were well-organized, and there were no pathological changes in the lamina propria of the mucosa (Figure 3 and Figure 4). Additionally, the reticular fibers shown in Figure 5 exhibited a compact arrangement. In contrast, the CMG and TMG groups displayed loosely woven bundles of collagen fibers, with a noticeably larger area of the intestinal wall occupied by the submucosa (Figure 3). Compared to the study groups, the control group showed a significantly higher number of clearly organized reticular fibers within the villi core, mucosal dermis lamina, and submucosa (see Figure 5).

### 3.2. Morphometry

**Figure 6:**
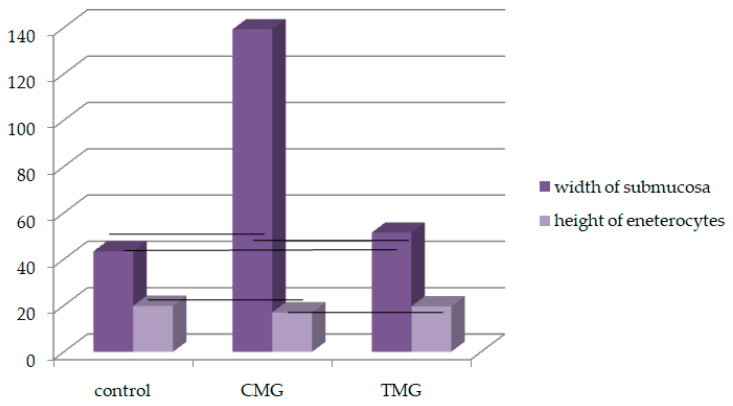
Morphometric analysis between examined groups, black lines: *p* < 0.05.

This study’s results on the morphometric analysis of the submucosal thickness of the small intestine revealed that the thickest submucosal layer was observed in the CMG group, while the thinnest was in the control group, and the values showed significant differences. Additionally, significant variations were noted in the submucosal width between the CMG and TMG groups. Measurements of epithelial height indicated that the highest epithelial height was observed in the control group and the lowest in the CMG group. Furthermore, there were notable differences in epithelial height between the CMG and TMG groups (Table 1, Figure 6).

### 3.3. Immunohistochemistry—Digital Analysis

**Figure 7:**
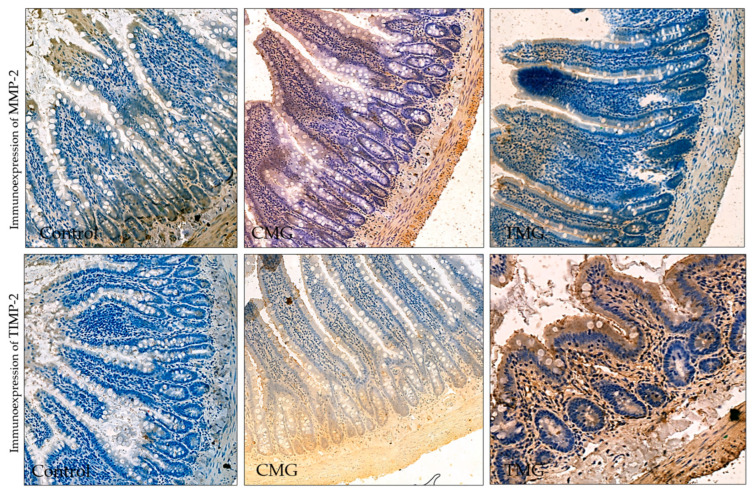
Immunoexpression of MMP-2 and TIMP-2 in the small intestinal wall.

In terms of MMP-2 and TIMP-2 immunolocalization, the values can be arranged in the following series: control group < CMG group < TMG group. The results of immunohistochemical reactions showed that the largest percentage of the area occupied by MMP-2 and TIMP-2 was in the TMG group. Both MMP-2 and TIMP-2 immunoexpression differences between the groups were statistically confirmed (*p* < 0.05) (Figure 7, Table 2).

**Figure 8:**
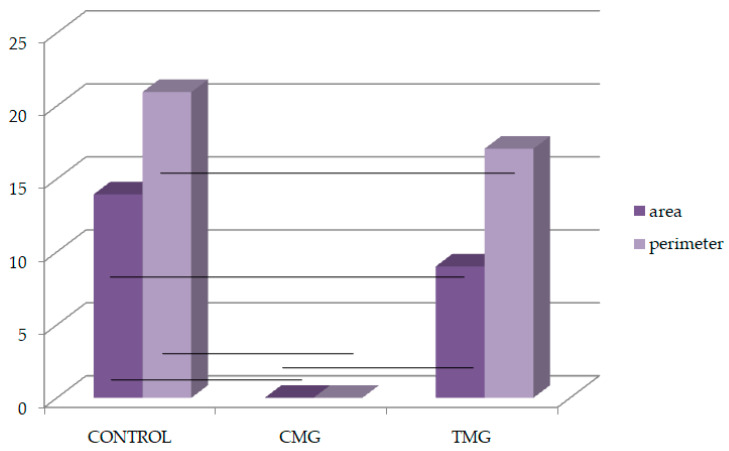
Area and perimeter occupied by MMP-2 (%); black lines: *p* < 0.05.

**Figure 9:**
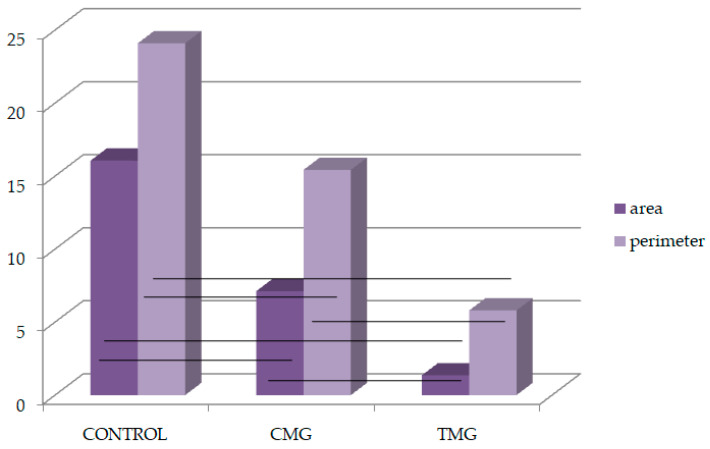
Area and perimeter occupied by TIMP-2 (%); black lines: *p* < 0.05.

Analyzing the results obtained for the perimeter of sites with immunopositive immunohistochemical reaction, it was found that considering MMP-2, the longest perimeter line was recorded in the control group and the shortest in the CMG group. The differences between all groups were confirmed statistically. Considering the perimeter of the TIMP-2 expression, the largest values were for the TMG group, while the smallest values were for the CMG group. The differences between all groups were also significant. The largest difference between TIMP-2 and MMP-2 was observed in the TMG group, and it was 21.82 (Table 2, Figure 8 and Figure 9).

Analyzing the results obtained on the balance between MMP-2 and TIMP-2 showed significant differences in the area and perimeter of their immunoexpression. The most disturbed MMP-2/TIMP-2 balance, considering the immunopositive area, was observed in the TMG group. Comparing the results on MMP-2 and TIMP-2 immunoexpression in terms of perimeter, it showed that the greatest difference between MMP-2 and TIMP-2 was seen in the TMG group, confirming that TAC disrupted the MMP-2/TIMP-2 balance the most (Table 3).

Additionally, digital image analysis of the intensity of MMP2 and TIMP2 immunoexpression in each group showed that the area with the strongest immunoexpression was the highest in the TMG group. In the TMG group, immunoexpression at the 3+ level for TIMP2 was 4.58% (Table 4). In the case of the control group, the highest percentage of MMP2 was for proteins with very low intensity, while for TIMP2, 100% of the area was occupied by structures with very low intensity. The most diverse group in terms of immunoexpression intensity was the CMG group. The highest percentage was for structures with 2+ intensity, at 67.27%. In the case of TIMP2, the entire area was negative.

## 4. Discussion

The gastrointestinal tract is the most complex of all the body’s organ systems, with numerous structures and functions, as well as various signaling molecules involved. It is responsible for digesting food and absorbing nutrients, electrolytes, and water in a tough, diverse microbiota-filled environment. Our gastrointestinal tract has specialized structures that support these intricate processes [27,28,29,30,31]. The morphology of the gastrointestinal tract can be disrupted by factors such as immunosuppressive drugs, which are vital for organ transplant patients. These morphological changes can affect different layers of the intestinal wall and the expression of metalloproteinases, which play a role in the organization of the intercellular matrix [8]. The main goal of this study was to examine the morphology of the small intestine in rats long-term treated with panels of calcineurin inhibitor (CNI)-based immunosuppressive drugs and to investigate their effects on the localization of MMP-2 and its inhibitor through immunohistochemistry.

The conducted experiment confirms the negative effect of CNIs on the morphology of the small intestinal wall. The results show that these drugs disrupt the structure of collagen fiber bundles. In the control group, the collagen fiber bundles in the submucosa were properly organized, and there were no pathological changes in the dermis of the mucosa. In contrast, the CMG and TMG groups showed loosely woven bundles of the fibers. In terms of reticular fibers, there were significantly more of them in the control group compared to the study groups. In the control group, reticular fibers showed a compact arrangement, and there were more of them in the area of the villi core, the dermis lamina of the mucosa, and the submucosa compared to the CMG and TMG groups. These results confirm that both CsA and TAC negatively affect the morphology of the intercellular matrix fibers of the small intestinal wall.

Apart from changes in shape, the results also revealed changes in the size of the small intestine. In the groups studied, CMG and TMG, the submucosa widths were significantly larger compared to the control group.

It is possible that CNIs (calcineurin inhibitors) may disrupt the ratio of MMP-2 to TIMP-2 expression, leading to disorganization in the production of intercellular matrix components, such as collagen fibers. This could result in an increase in the width of the submucosa, which contains connective tissue. It is important to note that both CsA (cyclosporine A) and TAC (tacrolimus)-based treatments undoubtedly impact the measurements related to the submucosal layer of the small intestine.

Additionally, the analysis revealed that the tallest epithelium was observed in the control group and the shortest in the CMG group, which was confirmed through statistical analysis. Moreover, there were significant differences in epithelial height between the CMG and TMG groups.

There is limited information on how immunosuppressive drugs directly affect the expression of matrix metalloproteinase 2 (MMP-2) in the small intestine. MMP-2 is known to play a role in tissue remodeling and repair, and its expression can be influenced by various factors, including immune system activation. These drugs can have complex effects on different cell types and tissues in the body. While there is no direct evidence of the effect of immunosuppressive drugs on MMP-2 expression in the small intestine, it is possible that these drugs may indirectly affect MMP-2 by suppressing immune cells and the inflammatory response. Inflammation can trigger MMP-2 expression, and by reducing inflammation, immunosuppressive drugs may potentially reduce MMP-2 levels in the small intestine [32].

The immunohistochemical studies conducted for this research revealed an imbalance between MMP-2 and its inhibitor TIMP-2. The immunoexpression of TIMP-2 was found to be increased in the groups under study. When considering the immunolocalization area of MMP-2 and TIMP-2, the values can be ranked in the following order: control group < CMG group < TMG group. The results of the immunohistochemical reactions showed that the TMG group had the highest percentage of the area occupied by both MMP-2 and TIMP-2, as well as the greatest difference in immunoexpression between the two proteins. Statistically significant differences in immunoexpression were confirmed for both MMP-2 and TIMP-2.

Analyzing the perimeter of sites with immunopositiveimmunohistochemical reactions, it was found that the control group had the longest perimeter line when considering MMP-2, while the CMG group had the shortest. Statistically significant differences were confirmed for all groups. For the perimeter of the TIMP-2 expression, the largest values were observed for the TMG group, while the smallest values were found in the CMG group. Additionally, the TMG group exhibited the largest difference between TIMP-2 and MMP-2. These findings align with those of other researchers. Comparing the two drugs of the CNIs group, it was noted that TAC causes greater changes in organ morphology than CsA, not only in the kidney and liver but also in the intestine [18,33].

Data on the effects of CNIs on the immunoexpression of metalloproteinases and their inhibitors in the small intestinal wall are limited. However, side effects of CNIs on metalloproteinases in other organs have been described. For example, Ha and Mun (2012) described the adverse effects of CsA on vascular structure by upsetting the balance of specific endopeptidases, leading to an increase in the activity of certain metalloproteinases and a decrease in others [34]. Additionally, Waller et al. (2005) showed that the use of CNIs in combination with MMF reduced certain expressions compared to monotherapy with the mentioned drugs [35].

A study by Nazemisalman et al. (2019) showed that CsA affected the secretion of MMPs and TIMPs, indicating greater degradation of ECM. Furthermore, an analysis of the results obtained on the balance between MMP-2 and TIMP-2 showed significant differences in the immunoexpression of both proteins. Our results suggest that the TAC-based drug panel most disrupts the MMP-2/TIMP-2 balance in the small intestinal wall in rats, likely due to its absorption in the gastrointestinal tract and metabolism by liver enzymes. The results were also confirmed by digital image analysis of tissue segmentation, showing that tacrolimus most disturbs the expression of the proteins studied and the balance between them [36].

## 5. Conclusions

The research conducted in this study is unique because there is limited information available on the direct effects of immunosuppressive drugs on the expression of MMP-2 and its inhibitors in the small intestine. Additionally, this study involves three drugs instead of one, which accurately reflects the panel of drugs used in organ recipients. The results indicate that immunosuppressive drug panels containing calcineurin inhibitors (CNIs) have a negative impact on the morphology and morphometry of the small intestinal wall. These drugs disrupt the MMP-2/TIMP-2 balance. Both CsA and TAC interfere with the synthesis of intercellular matrix components in the connective tissue of the small intestine. Furthermore, tacrolimus appears to disrupt the MMP-2/TIMP-2 balance in the small intestine the most, as the results show the highest difference between MMP-2 and TIMP-2 expression. AI-based tools provide a reliable analysis of tissue samples, which represents an exciting approach for future histopathological studies. However, the results of the analyses generated by these tools need to be verified by specialists.

## Figures and Tables

**Table 1:** Morphometric analysis of the small intestinal wall (µm). N—number of measurements; n—number of individuals; AM—arithmetic mean; SD—standard deviation; Med.—median; CV—coefficient of variation, * *p* < 0.05.

Width of Submucosa			
	Control group	CMG	TMG
	n = 6	n = 6	n = 6
N	30	30	30
AM ± SD	42.95 ± 8.89	134.72 ± 20.94 * ^vs. control^	51.87 ± 9.38 * ^vs. control vs. CMG^
Med.	43.45	139.3	51.5
CV	20.71	15.54	18.09
**Hight of Enterocytes**			
	Control group	CMG	TMG
	n = 6	n = 6	n = 6
N	60	60	60
AM ± SD	20.17 ± 2.056	17.19 ± 1.93 * ^vs. control, vs. TMG^	19.52 ± 2.24 * ^vs. CMG^
Med.	19.89	17.07	19.67
CV	10.21	11.23	11.49

**Table 2:** Descriptive statistics for area and perimeter occupied by MMP-2 and TIMP-2 (%). N—number of measurements; n—number of individuals; AM—arithmetic mean; SD—standard deviation; Med.—median; CV—coefficient of variation, * *p* < 0.05.

	Control	CMG	TMG
		**MMP-2**	
		**Area**	
N	804	1285	765
AM ± SD	311.50 ± 5813.075	346.88 ± 4779.96 * ^vs. control^	362.63 ± 642.18 * ^vs. control, CMG^
Med.	13.94	3.44	9.004
CV	1866.11	1377.95	1770.44
		**Perimeter**	
N	804	1285	765
AM ± SD	75.36 ± 396.506	47.46 ± 360.61 * ^vs. control^	70.81 ± 463.68 * ^vs. control, CMG^
Med.	20.95	11.55	17.07
CV	526.14	759.71	654.746
		**TIMP-2**	
		**Area**	
N	733	456	86
AM ± SD	333.98 ± 6015.93	749.34 ± 1468.91 * ^vs. control^	1693.01 ± 1512 * ^vs. control, CMG^
Med.	16.06	7.12	1.36
CV	1801.76	1950.75	893.21
		**Perimeter**	
N	733	456	86
AM ± SD	77.69 ± 364.39	60.85 ± 539.32 * ^vs. control^	92.63 ± 611.87 * ^vs. control, CMG^
Med.	24.102	15.43	5.802
CV	469.02	886.21	660.49

**Table 3:** MMP-2/TIMP-2 balance in small intestine.

	Control	CMG	TMG
**Area**			
MMP-2 (%)	311.50	346.88	362.63
TIMP-2 (%)	333.98	749.34	1693.01
TIMP-2-MMP-2 (%)	22.48	402.46	1330.38
**Perimeter**			
MMP-2 (%)	75.36	47.46	70.81
TIMP-2 (%)	77.69	60.85	92.63
TIMP-2-MMP-2 (%)	2.33	13.39	21.82

**Table 4:** The intensity of MMP2 and TIMP2 immunoexpression in each group (%).

	Control		CMG		TMG	
	MMP2	TIMP2	MMP2	TIMP2	MMP2	TIMP2
	(%)	(%)	(%)	(%)	(%)	(%)
0	83.13	100	5.45	100	0	2.61
1+	6.98	0	20.91	0	0	26.8
2+	9.88	0	67.27	0	40.5	66.01
3+	0	0	6.36	0	59.5	4.58

## Data Availability

The raw data supporting the conclusions of this article will be made available by the authors upon request.
